# John Wickham’s New Surgery: ‘Minimally Invasive Therapy’, Innovation, and Approaches to Medical Practice in Twentieth-century Britain

**DOI:** 10.1093/shm/hkw074

**Published:** 2016-10-06

**Authors:** Sally Frampton, Roger L. Kneebone

**Keywords:** surgery, innovation, radiology, technology, minimally invasive

## Abstract

The term ‘minimally invasive’ was coined in 1986 to describe a range of procedures that involved making very small incisions or no incision at all for diseases traditionally treated by open surgery. We examine this major shift in British medical practice as a means of probing the nature of surgical innovation in the twentieth century. We first consider how concerns regarding surgical invasiveness had long been present in surgery, before examining how changing notions of post-operative care formed a foundation for change. We then go on to focus on a professional network involved in the promotion of minimally invasive therapy led by the urologist John Wickham. The minimally invasive movement, we contend, brought into focus tensions between surgical innovation and the evidence-based model of medical practice. Premised upon professional collaborations beyond surgery and a re-positioning of the patient role, we show how the movement elucidated changing notions of surgical authority.

## Introduction

In contemporary society increasing value is attached to the term ‘innovation’ and the concept of new, radical change that underlies it. The prominence and prevalence of ‘innovation’ in political rhetoric is reflective of the almost entirely positive connotations that have come to be associated with the term.^1^ But emerging innovations have rarely been accepted unquestioningly. Rather, innovation is a complex, contested and lengthy process, not simply the invention and introduction of ‘better’ products and services. In medicine especially, new procedures, technologies and theories have often triggered concerns about the risks they might bring, especially to the patient, and medical historians have been attentive to the interplay between risk and innovation.^2^

‘Minimally invasive therapy’ is one innovation nonetheless, which demands further exploration. Also known colloquially as ‘keyhole surgery’, the term ‘minimally invasive’ was coined in 1986, and ‘minimally invasive therapy’ in 1989 by urologist John Wickham to describe a range of procedures that required making only very small incisions, or sometimes no incision at all, to treat diseases which previously would have required ‘open’ surgery (often necessitating a large incision).^3^ Its introduction represented a major change in British medical practice. Surgeons of today view the impact of the minimally invasive movement as seismic, fundamentally altering surgical practice and the attendant skills needed to perform procedures. In many spheres of surgery open operations are now a rarity.^4^ This sea-change in surgery begs the question of how the movement emerged and what exactly was driving change. But beyond the contemporary ramifications, the advent of minimally invasive practice also offers a case study through which to critically examine the process of surgical innovation in the late twentieth century.

This paper first puts the introduction of minimally invasive therapy in Britain into context by examining surgical norms during the nineteenth and twentieth centuries, particularly those relating to incision size and post-operative care. The incision is one of the most critical aspects of an operation and its presence is integral to the definition of surgery—the meaning of which is inextricably tied up with the notion of physical intervention. Yet it is not often the subject of critical examination by medical historians, treated instead as an unquestioned essential of the surgical process. As we show, the large incision of open surgery—a staple of medicine from the late nineteenth century and an iconic emblem of the power of the profession—came to be re-framed as unnecessary and even amounting to iatrogenic injury during the late twentieth century. This was, we suggest, related to broader changes in medical culture and disease patterns in the post-war period which led a new generation of surgeons to begin challenging long-standing tenets of surgery with new practices.

We then go on to focus upon the urologist John Wickham who, as well as being responsible for coining the phrase and introducing numerous minimally invasive procedures in his own field, was acutely aware from the outset of the wider implications of this new approach to surgery. Wickham formed the Society for Minimally Invasive Surgery—renamed the Society for Minimally Invasive Therapy (SMIT)—in 1989. Through it he sought to build a coherent theoretical framework around the technical innovations that were being developed by himself, his colleagues, and others across Europe and America. Detailing how both personal motivations and the professional culture he was part of led him to formulate innovations for traditionally invasive procedures of the urinary system, we use the case study of Wickham to suggest significant and broader aspects to this shift in surgical practice. We argue that these procedures forcefully demonstrated an incompatibility between the practically-based innovations Wickham and colleagues were developing, and the, by then prevalent, evidence-based model of medical practice. We then examine the implications of minimally invasive therapy for professional and organisational structures in medicine, looking at how the Society for Minimally Invasive Therapy framed a set of procedures as a challenge to traditional notions of professional hierarchy in medicine.

This paper draws heavily upon the oral histories of Wickham, those he collaborated with and other individuals who played significant roles in the move towards minimally invasive practice. This project originally set out to trigger recall of events which happened during the 1980s and 1990s, a fast-moving and unstable moment in British surgical history, by those who were directly involved. Examination of the contemporary literature took place alongside a series of individual interviews with key players. This was supplemented by group interviews in which we hoped that discussion would stimulate collective recollection. These built upon previous work by Roger Kneebone and Abigail Woods, which used simulation-based re-enactment to recapture both technical and social practices in surgery.^5^ Our aim was to capture memories of events, practices and explorations that were never published, and which therefore do not necessarily form part of the orthodox documentary canon. Over two years, a series of extended and detailed interviews focused upon Wickham and his colleagues were recorded.^6^ We aimed to capture multiple perspectives, those of surgeons, nurses, radiologists and instrument manufacturers, reflecting a key characteristic of minimally invasive practice at the time—its multidisciplinary nature. These testimonies, collected between 2012 and 2014, are by no means comprehensive; they include only a small number of the individuals involved in the move to minimally invasive practice. But the interlinked histories of Wickham, his collaborators and contemporaries, give a strong sense of the changes that were occurring and which underpinned minimally invasive therapy. Thus the article provides a thick description of Wickham’s experience, contextualising it within the longer history of surgery. It uses the case study of Wickham to show that individual experience can evocatively illustrate an aspect of the history of surgery which has been thus far been little historicised, being confined mostly to the work of Grzegorz S. Lityński and Lawrence Rosenberg and Thomas Schlich.^7^

By taking this approach, this article elicits the British experiences of minimally invasive therapy. The shift towards a coherent minimally invasive movement was a transnational one and developments in France, Germany and North America especially were critical to British practitioners, including Wickham. Some of these developments will be explored below. But drawing out structures and values specific to Britain, such as the politics of the National Health Service, will demonstrate how the assimilation of minimally invasive techniques into practice was shaped at a national level.^8^

## ‘Nothing Like Large Enough’ Surgical Incision Size and Post-operative Care in the Nineteenth and Twentieth Centuries

In modern surgery two values have often co-existed. One is a drive among surgeons to expand the applicability and usefulness of surgery across the bodily landscape. In the early nineteenth century, British surgeons performed a wide array of operations from amputation and lithotomy to complex reconstructive operations of the nose.^9^ But major surgery became increasingly possible during the middle of the nineteenth century, as new allied technologies—anaesthesia, antiseptics and artery forceps in particular—enabled surgeons to increasingly experiment with procedures that went further into the body. During the second half of the nineteenth century, organs of the abdomen and pelvis (especially the ovary, the uterus and the kidney) became recognised sites for major operations, as surgeons rapidly advanced upon the internal cavities. By the late 1880s optimism prevailed among surgeons that they had vastly expanded their territory across the body, bringing the internal organs into the surgical view and creating a legacy of safe, painless surgery.^10^

However, the hunt for new surgical possibilities had long been tempered by a second value: that surgeons should inflict the least possible trauma upon patients. Whether motivated by humanitarian concerns or by fears regarding professional reputation, surgeons of the eighteenth and nineteenth centuries were vocal about the need for operations to be performed only when absolutely necessary: in the late 1780s, John Hunter instructed his students that the necessity for operations at all was ‘in truth the Defect of Surgery’.^11^ Major operations carried a high risk of death or disease and the moral basis of surgery rested upon the notion that operations should be the last resort when all else failed. Increasingly, the practitioners considered most skilful were those who were able to use their knowledge of pathology to treat or cure without recourse to the knife.^12^ This philosophy of practice was tested as surgery expanded into new areas of the body and surgeons remained frequently open to charges of ‘over-operating’ in the late nineteenth century. A tension in values saw the profession collectively sanction the use of an increasingly diverse range of operations, while continuing to police against the unnecessary use of surgery.^13^

The question of incision length often formed part of these concerns. In the nineteenth century ovariotomy—the removal of diseased ovaries—was the testing ground for abdominal surgery. First performed in 1809, by the 1840s it had become the first established operation to involve opening the peritoneal cavity, an act which had previously been thought to lead to the inevitable death of the patient.^14^ The growing success of the operation over the next decades showed this not to be the case and the operation was hailed as opening a new era of surgery. Initially ovariotomy was thought to require large incisions (up to twelve inches long) to ensure a high level of visibility in the abdominal cavity and to enable surgeons to check for adhesions and complications.^15^ These large incisions made early ovariotomists targets of derision and disgust, allegedly earning them the unsavoury nickname of ‘belly-rippers’ from their critics, who played upon the operation’s unpalatable associations with human vivisection.^16^

During the nineteenth century, ideas about incision length often altered with changing trends in surgery. As Thomas Schlich has shown, a growing interest in complex, conservative procedures in abdominal surgery towards the end of the century meant small incisions were frequently used to perform procedures such as enucleation.^17^ Moreover, individual surgeons often had to vary incision length depending on the individual factors of each case they were presented with. Nonetheless, by the early twentieth century, it had been reliably demonstrated that large incisions could be safely made into the peritoneal cavity. For the profession, the large incision was the physical embodiment of the wonders of modern surgery; the hard-won prize of surgeons who had risked their reputations by going into the human abdomen. Procedures involving large incisions became an accepted—if unpleasant—part of the surgical repertoire. Indeed, they would come to characterise it: one of the most memorable scenes from the hugely popular medical comedy film *Doctor in the House* (1954), starring Dirk Bogarde, is the arrival of imposing chief surgeon Sir Lancelot Spratt (played by James Robertson Justice) to conduct a bedside examination with his students. ‘Nothing like large enough’ Spratt tells one of his students irritably, after the young man makes a small mark for where an incision might be made, ‘keyhole surgery, damnable, couldn’t see anything … like *this*’ Spratt declares, before drawing a long line across the unfortunate patient’s abdomen.^18^ Caricature though this might be, playing as it did on the masculine bravado of the operating surgeon and somewhat exaggerating the profession’s enthusiasm for the largest possible incision, between the 1880s and the 1980s large incision procedures were the norm in abdominal surgery and a stability in technique prevailed in many operations.^19^

Voices of dissent did challenge the status quo. In 1934 the Philadelphia surgeon James W. Kennedy made a rallying call for a gentler kind of surgery in an essay for the *American Journal of Surgery*, emotively titled ‘Tragedies of the Abdominal Incision’. In it Kennedy drew attention to the number of surgical deaths that could be attributed to infected wounds and other incision-related ailments, claiming that abdominal incisions were often twice the length necessary. Kennedy lamented ‘living in a surgical era where the blow of surgery is taken from the operator and placed upon the patient’.^20^ In general, surgeons wanted incisions that offered the best access with the least amount of injury to the body or post-operative complications for the patient. However, most surgical meditations on the subject did not frame the issue in terms of an incision’s level of invasiveness or explicitly in terms of prioritising the patient experience.^21^ The idea of a small ‘keyhole’ incision was, for the most part, treated with caution in the first half of the century. One of the earliest references to the phrase in the medical press was made by the British surgeon Alfred Pearce Gould, author of the popular *Elements of Surgical Diagnosis*, who warned in 1911 that surgeons lacking experience in abdominal surgery should ‘not try to do difficult operations through a keyhole; wait for that until you have learnt to do them through an open door’. While attempting to minimise incisions was thought possible, it was seen to require a degree of technical proficiency that all but the most highly-versed in abdominal procedures would be lacking.^22^

This immutability of practice was sustained by a professional culture which erred on the side of conservatism. One of our interviewees, Mr Chris Russell, who qualified in 1963 and is today retired after a distinguished career as an upper gastrointestinal surgeon, remembered the atmosphere at the Middlesex Hospital in the mid-decades of the century, when he undertook surgical training with Sir Cecil Murray (1910–1991). During the Second World War Murray had been a lieutenant colonel in the Royal Army Medical Corps and for Russell, the surgeons of Murray’s generation had: ‘ … an element of military properness … [it was] very authoritarian. If you’d been to public school you were used to that structure.’^23^

For young trainees like Russell, who would later become an early advocate of minimally invasive practice working alongside John Wickham, there were obvious benefits to this structure; a high degree of camaraderie prevailed, as surgeons from across the professional strata worked together within close knit surgical ‘firms’, which provided continuity, stability and support while also sustaining an implicit hierarchy. However, this authoritarian structure left little scope for questioning established norms. This was perhaps most evident in the attitude of senior surgeons to patients’ post-operative recovery. A lengthy period of convalescence was both expected and encouraged after major operations involving large incisions, and surgeons commonly insisted upon protracted periods of bed rest and subsequent avoidance of work following a procedure.^24^ Toward the end of the 1950s, this convention began to be critically questioned. New research suggested that determination of convalescence time was based less on empirical evidence than on the ‘impressions and vague concepts’ of individual physicians and surgeons.^25^ Increasingly it seemed that those practitioners with unexamined standard aftercare periods risked draining hospital resources.^26^ Such investigations chimed with broader trends in British medicine. The social medicine movement, which formalised with the establishment of the Social Medicine Research Unit in 1948, encouraged doctors to collate and compare epidemiological data so as to evidence their rationale for treatment plans.^27^ The financial efficiency and resource management of the still young National Health Service was also undergoing a period of scrutiny, culminating in the Guillebaud Report of 1956.^28^ Subtle pressure was exerted upon surgeons to reconsider the length of hospital stay they prescribed their patients.^29^

Disease patterns were also changing and placing greater demands on surgeons. As tuberculosis and other infectious diseases went into decline, localised ‘surgical’ diseases such as cancer became the focus, with both medical professionals and patients expressing increasing dissatisfaction with the radical operations used to treat it.^30^ Speaking in 2012, the surgeon Mr David Rosin, who performed the first laparoscopic cholecystectomy in England in 1990, recalled the impact of this ethos upon practice. Like Russell Rosin had found surgery at a standstill during the early days of his surgical training. As a student in the 1960s Rosin recalled that ‘surgery at the time … had not changed really for eighty years’.^31^ But as he worked his way up to the position of senior registrar in the 1970s, he perceived a noticeable shift in surgeons’ practices, towards a ‘less mutilating surgery, especially in cancer’.^32^ Operations like radical mastectomy for breast cancer—a first-line treatment for the disease since the nineteenth century—met increasing resistance as doubts grew over the justification for using such an invasive procedure on the basis of it eradicating future risk of recurrence. The operation soon became a target of criticism for the fledgling breast cancer activism movement.^33^ The movement was itself indicative of a shifting patient identity in the mid-decades of the twentieth century. As Alex Mold has shown, by the 1960s patients were finding new and authoritative means of challenging the paternalistic healthcare of the NHS, and organisations such as the Patients Association, established in 1963, began to re-model the patient identity from that of passive recipient to one of active, informed consumer.^34^

By the time this new generation of post-war surgeons attained consultant rank, a groundswell of medical opinion (within and beyond surgery) was challenging accepted surgical dogma with some individuals playing a crucial role in bringing about conceptual change. One of these was John Wickham.

## Imagining the ‘new surgery’: John Wickham, percutaneous nephrolithotomy and the minimally invasive movement

John Wickham was born in Chichester in 1927 and qualified in medicine in 1955. Undertaking his early training at St Bartholomew’s Hospital, Wickham would remain at the institution for most of his career, eventually combining his role there as consultant urologist, which began in 1968, with an appointment heading up the academic unit of the Institute of Urology, which served London’s three specialist urology hospitals, St Peter’s, St Paul’s and St Philip’s.^35^ Consistent with the experiences of Russell and Rosin, the young Wickham became quickly frustrated to find general surgery was ‘static’ and resistant to change. An early experience as a house officer in neurosurgery, an uncommon rotation for a young doctor during the mid-decades of the century, would prove one of the greatest influences on his career.^36^ Neurosurgery, where the smallest bleed could wreak havoc with the patient’s brain function, necessarily involved employing the highest degree of delicacy and precision. For Wickham the difference in technique between neurosurgery and general surgery was astonishing. Upon his return to general surgery after the completion of his house duties he found himself appalled by the ‘great slashes and blood and guts everywhere’. His personal antipathy to large incisions, illuminated by his experience of neurosurgery, proved to be a powerful motivating factor in his later innovations. As he recollected in 2012, ‘when I came back to ordinary surgery I was just disgusted … I mean if you could be that delicate in neurosurgery, why couldn’t [general surgery] be the same?’.^37^

Wickham eventually decided to train in urology, intrigued by the rapid developments that were occurring in kidney dialysis and transplantation. But one of the bread-and-butter areas of work for urologists remained the treatment of renal calculi (kidney stones). These were usually dealt with by making a large open incision in the flank to reach the kidney, which was opened to remove the stones and then repaired—often inflicting considerable trauma in the process. The procedure—the use of a large incision to take out what were frequently minuscule stones—only highlighted to Wickham the excessiveness of open surgery in comparison to the disease being treated. As he recalled: ‘I was fed up taking out tiny little stones and going to the patient the following day and saying “look we got your stone out!” with a socking great gash … which seemed totally daft’.^38^

Wickham was, of course, not the first to suggest there might be a different way of doing surgery. Since the late nineteenth century endoscopic instruments—flexible tubes with a light source attached that could be inserted into the body—had been used to inspect internal organs and, from the early twentieth, to take tissue biopsies for diagnosis. The 1930s had seen a noticeable development with the transition of the endoscope from an exploratory tool to a therapeutic one, as a handful of European doctors began to perform minor surgical procedures of the abdomen via tiny ‘laparoscopic’ incisions.^39^ One of the earliest examples was German physician Carl Fervers, who in the 1933 began to cauterise abdominal adhesions by this method.^40^ By the 1980s surgeons in North America, Germany and Japan were attaching miniscule video cameras to endoscopic instruments. This latter development allowed surgeons to view the internal body on a television screen rather than having to look directly through the endoscope, enabling surgeons freer movement and increased visual clarity through magnification.^41^

In the late 1970s, however, as individuals like John Wickham pondered possible ways of minimising the trauma of surgery, the greatest impact was perhaps not from European laparoscopy but rather the fledgling field of interventional radiology. Prior to the 1970s, the work of radiologists had mainly been restricted to interpreting radiographs. The use of radiological imaging as a therapeutic tool was very limited.^42^ New technologies considerably expanded the specialty; ultrasound, computerised tomography (CT) and Magnetic Resonance Imaging (MRI) together provided unprecedented scope for making images of the internal body. This in turn boosted the standing of radiologists. Relocating from the darkened rooms of hospital basements to the wards and clinics of the hospital, radiologists ascended both physically and professionally.^43^ Dr Michael Kellett, who qualified in 1969 and went on to work closely with John Wickham, remarked in one interview: ‘we suddenly came up a step and realised that we were perhaps indispensable after all’.^44^

This levelling of access to the professional playing field (although not a flattening—surgeons were still accorded a higher occupational status than radiologists), proved critical in allowing an exchange of ideas between Wickham and Kellett. Having first met in the 1970s when Wickham was a urology registrar at St Peter’s, Wickham and Kellett began exchanging ideas that led to the development of a new procedure for renal calculi in 1979. Influenced by developments in German urology and blending technologies and methods from radiology and surgery, the percutaneous nephrolithotomy was introduced by Wickham and Kellett to the British medical community in 1981 in an article in the *British Medical Journal*:^45^

Small dilators were introduced over a guide wire through a nephrostomy tube into the renal pelvis and a catheter inserted. The track was dilated in stages and two days later the nephrostomy tube was removed and a cystoscope introduced into the interior of the kidney. A stone basket was introduced down the operating channel of the cystoscope and manoeuvred to secure the stone; the cystoscope, stone basket, and stone were then removed.^46^

Percutaneous nephrolithotomy involved a protracted operating period shared between the radiologist and urologist, with Kellett responsible for the gradual dilatation of the tract, before the final operating stage was carried out by Wickham a couple of days later. Thus, the method involved a significant change in the temporal horizon of surgery, where a longer surgical procedure was offset by a recovery and convalescence that was expected to be much shorter. While future innovations by Wickham and Kellett would reduce procedure time, this transition was a defining trait of the minimally invasive movement and a controversial one too: laparoscopic procedures would later go on to earn the epithet ‘foreveroscopy’.^47^

This introduction of a lengthier, more technologically reliant procedure could be construed as an expansion of practitioners’ control over patients’ bodies. The incorporation of technology upon modern medicine has often been perceived as being in tension with more humanistic concepts of medical care; interventional and diagnostic technologies can be viewed as invasive, impersonal and alienating, physically distancing the patient from the practitioner and further diminishing the significance of the patient’s own experience.^48^ In surgery, the extension of a patient’s time under anaesthesia, during which the person is rendered an unconscious body, might be understood similarly. However, such a reading omits the rather tangled connections between corporeality, technology and surgery that were being negotiated in percutaneous nephrolithotomy. For Wickham and Kellett, it was crucial that their new methods were framed as patient-centred rather than an assertion and expansion of medical authority. For Wickham, what was important about minimally invasive procedures was that they were a means of ‘minimising renal damage … iatrogenic damage’.^49^

Iatrogenesis—a term Wickham used both then and in recent interviews—was a politically loaded concept at the time percutaneous nephrolithotomy was being introduced. ‘Iatrogenic’ had long been used by doctors to refer to illness or bodily damage that was the result of medical intervention.^50^ But during the 1970s it became more popularly known through the work of Ivan Illich. In 1975 Illich had published *Medical Nemesis* (also known as *Limits to Medicine*) in which he launched a scathing attack on what he perceived to be the full and devastating extent of ‘the sick-making powers of diagnosis and therapy’.^51^ Through Illich’s work, iatrogenesis was re-defined as more than an unfortunate consequence of medical intervention. Instead it came to convey the detrimental physical and social effects of western medicine.

Wickham used the concept of iatrogenesis to buttress his argument that surgeons needed to find alternatives to open surgery that would be less traumatic for patients. Increasingly, for Wickham, it was not simply about individual changes in technique, but about a fundamental reform of practice. In a leading article in the *British Medical Journal* in 1987, self-assuredly titled ‘The New Surgery’, Wickham described the endoscopic procedures beginning to be rolled out across the specialties, from vascular surgery to neurosurgery. Unifying these procedures under the name ‘minimally invasive surgery’, although also using the colloquial term ‘keyhole surgery’ (discussed further below)—Wickham characterised the movement as a patient-centred one:

Surgeons applaud large incisions and denigrate ‘keyhole surgery.’ Patients, in contrast, want the smallest wound possible, and we at Britain’s first department of minimally invasive surgery are convinced that patients are right. What makes patients ill after an operation is the iatrogenic damage that surgeons have inflicted in achieving their technical aim.^52^

Thus Wickham posited his innovations as not simply a new set of procedures but a new system of surgery. This was premised upon notions of both cultural and technological change. His assertion that the ‘patients are right’ reflected the increasing challenges that were being made to paternalistic health care.^53^ It was this overt repositioning of the patient-consumer’s preferences over the surgeons’ which distinguished this debate about incision size from those which had occurred previously. The new system also saw a transition from one sensory framework to another, as a set of skills different to those of open surgery were prioritised. Surgeons would be guided by what they saw rather than what they felt, and that seeing was mediated in quite a different way—usually through a lens or camera—rather than direct vision.^54^ For Wickham, a more technologised surgery equated with a more patient-friendly form of surgery and away from an older style of practice. As he would later describe it ‘… this business of needing to get your hands in to feel something … that’s about 1890 I would have thought, or 1850’.^55^ This characterisation somewhat simplifies the historical record; nineteenth-century surgeons, particularly specialists in abdominal procedures, were extremely cautious about the rough handling of the internal organs by surgical hands and feared injuring them through their actions.^56^ In his desire to distance himself from the corporeality of surgery, Wickham was not the first: historian Christopher Lawrence has traced a long history of surgeons working hard to shake off ‘undesirable associations’ with the manual aspects of their work, which suggested intellectual inferiority to physicians.^57^ Wickham’s depiction nonetheless speaks to the centrality of palpation and touch as tenets of surgical practice at the end of the nineteenth century. This is amply demonstrated in the debate which arose around surgical gloves at the turn of the century. As Thomas Schlich has shown, their lengthy and complex negotiation into surgical practice was in part due to concerns that they would cause ‘impairment of touch, finger mobility and manual dexterity’.^58^

By embracing technology, Wickham was also re-asserting the importance of performance and craft in shaping surgery, pitching it as a technological enterprise rather than a purely scientific endeavour. For Wickham technologically heavy, minimally invasive surgery was associated with innovation, and the freedom to change practices through designing new instruments, re-defining existing ones, and blending and altering procedures. This approach was not, however, shared by all in the surgical community. As Wickham developed further procedures, growing tensions were revealed between Wickham’s prioritisation of creative invention in surgical therapeutics and prevailing norms of scientific medicine.

## Evidencing Surgical Innovation

With a growing impetus behind minimally invasive procedures in the 1980s, it was not long before percutaneous nephrolithotomy was itself replaced by a new procedure for the treatment of renal calculi—extracorporeal shock wave lithotripsy (ESWL). ESWL was a procedure administered by a complex machine, the ‘lithotripter’, a large device that was contained in a water bath into which anaesthetised patients were lowered. Once the patient was in position, focused shock waves were transmitted through the water to the area of the kidney where the calculi had been located by radiological imaging, causing disintegration of the stones and their eventual passing. The first procedure was performed by Munich urologist Christian Chaussy in 1980.^59^

On hearing of its successful use, John Wickham had been keen to bring the lithotripter to Britain and applied to the Department of Health and Social Security (DHSS) to fund a machine for St Peter’s hospital. Formed in 1968 from the ministries of Health and Social Security, the creation of the department considerably bolstered the place of health issues in Cabinet discussion.^60^ The 1960s and much of the 1970s saw the Department garner success in attaining increased financial resources for the NHS.^61^ But by the early 1980s, when Wickham petitioned the Department, there had been a fall in spending as the Thatcher government sought to reduce the expenditure of the NHS amidst moves to reorganise its management.^62^ The lithotripter cost in the region of one million pounds, and the department, perhaps unconvinced of the cost-effectiveness of the new technology, twice rejected Wickham’s application for funding. The Department eventually supported the installation of a lithotripter at St. Thomas’, a large NHS Hospital, in 1983, in an agreement which saw the machine jointly funded by the private healthcare company British United Provident Association (BUPA). Its installation was a testing ground for the type of public–private partnership that the Conservative Government increasingly encouraged in the National Health Service.^63^

Still determined to install his own lithotripter, Wickham eventually secured private funding and established the London Stone Clinic in Welbeck Street, at the heart of elite London medical practice, using the machine to treat both private and NHS patients.^64^ In 1985 Wickham and his team published the results of their first 50 ESWL cases. The results, if not electrifying, were at least promising: 34 patients showed radiological evidence of their stones fragmenting, with seven completely free of stone when discharged. With an average hospital stay of just 3.7 days afterwards, and an operative period which lasted on average 23.5 minutes, ESWL seemed to promise both a reduced aftercare period and complete non-invasiveness—a procedure for a ‘surgical’ disease which required no surgery at all. ‘With no deaths and minimal morbidity the procedure has been shown to be safe and effective and is universally accepted by patients’ the articled declared.^65^

A number of correspondents to the *British Medical Journal* responded critically to this conclusion, questioning whether the results could be deemed successful when only seven patients were stone-free. A later report of 1,000 cases from clinicians running the lithotripter at St Thomas’ Hospital showed a similarly moderate success rate: only 44 per cent of patients were stone free when assessed three to six months after follow-up.^66^ However, what prompted greatest debate on the pages of the journal were not the results per se but the research practices underlying the innovation. Neither centre had undertaken a randomised controlled trial (RCT) for ESWL. Wickham’s team at the Institute of Urology had instead produced a report comparing ESWL to previous ‘historical’ cases of percutaneous nephrolithotomy and open surgery dating back to 1972, concluding from these that ESWL proved the most effective and cheapest way to get rid of stones.^67^ Meanwhile at St Thomas’ efforts to conduct an RCT had been thwarted after the hospital’s proposal to do so was rejected by the DHSS. The Department deemed that a trial would not be in the patients’ best interest, when randomisation meant the difference between a non-invasive procedure and an invasive one. Researchers from St Thomas’ took to the pages of the *British Medical Journal* to complain that ‘there appears to be two standards for innovation in medicine: one which demands rigorous assessment of new drugs by proper trials; and a second which allows the introduction of expensive technology and new techniques on the strengths of descriptive reports’.^68^

By this time the randomised controlled trial was on its way to becoming the chief methodological tool for practitioners in scientifically assessing the effectiveness of therapeutics in many areas of medicine.^69^ Post-war the RCT began to be heralded with anticipation that it would lead to ‘antiquated attitudes [being] swept away and replaced by a rational medicine based on unbiased prospective randomized trials’.^70^ Thus, in part, its rise was connected with values that had motivated Wickham in his minimally invasive project: a break from conservative structures of medical authority which were thought to encourage unquestioning acceptance of norms in practice.

However technological innovations in surgery largely remained outside the domain of the controlled trial. As David Jones has shown in his study of the development of coronary artery bypass grafting (CABG) in the early 1970s, tensions were already apparent in the surgical community regarding the value of randomised trials within the field as a form of evidence. Unlike pharmaceutical therapeutics, many surgeons believed that the efficacy of a surgical procedure was quickly and visually evident to all involved without the need for a trial. Moreover, some surgeons argued that the RCT was simply unsuitable for surgery given the idiosyncrasies of each surgeon and each surgical case which made standardization difficult.^71^ Yet in the shadow of RCTs becoming the ‘gold standard’ for assessing new treatments, the relative imperviousness of surgery to the method put it increasingly at odds with other branches of medicine.^72^

Through ESWL Wickham and other commentators were further elucidating tensions between two different models for the creation and dissemination of new modes of practice. The backbone of the randomised controlled trial—the term coined for it in the late 1980s was ‘clinical equipoise’—was that the basis for any trial needed to rely on genuine uncertainty within the medical community to make it ethically justifiable.^73^ In the case of lithotripsy, the existence of that was a matter of contention. As Wickham positioned it, the benefits of lithotripsy were self-evident, not just to the practitioners but to the patient too:

The man on the Clapham omnibus with a stone in his kidney requiring removal needs no controlled trial to tell him that there is an order of magnitude of difference between having a 12-inch loin incision—one of the most painful in surgery—two weeks in hospital, and six weeks’ convalescence versus two to three days in hospital with a virtually painless procedure followed by immediate return to normal activity.^74^

The rejection of a randomised controlled trial for lithotripsy at St Thomas’ seemed to legitimate Wickham’s view that not only was lithotripsy a superior treatment, but that there was an inevitability to the spread of less invasive procedures before formal assessment, rendering the clinical trial unnecessary. Significantly this division appeared to be authenticated by the state in the DHSS’s rejection of the RCT. ‘I told them … these machines are coming!’ recalled Wickham in 2012 on his appeals to the Department for the lithotripter—although neither the obviousness nor inevitability of ESWL was apparent to his critics.^75^ This had an effect in real terms: Michael Kellett remembered a degree of hostility towards their lithotripsy practice, with doctors hesitant to refer patients to the centre, suspecting the lithotripter to be no more than ‘John Wickham’s plaything’,^76^ although referrals to the centre soon began to increase, as did the number of lithotripters in NHS practice.

The discordance between the randomised controlled trial and technological innovation would play out throughout the early history of the minimally invasive movement in Britain, supporting Jones’ thesis of the contestation of RCTs in surgery. It was an issue that was to become particularly controversial with the introduction of laparoscopic cholecystectomy (removal of the gallbladder though a number of small incisions). The first laparoscopic cholecystectomy had been performed by German surgeon Eric Mühe in 1985.^77^ The procedure was first performed in Britain in 1990. Its subsequent rapid diffusion throughout the profession signalled the entrance of minimally invasive techniques into general surgery. By the mid-1990s minimally invasive practice could no longer be seen as a fringe movement, but a force throughout the surgical sphere. Laparoscopic operations were being widely practised across surgery despite a lack of clinical evidence regarding their benefits. The lightning-quick uptake of laparoscopic cholecystectomy was soon a source of controversy, as a number of high profile cases, in which complications had followed laparoscopic procedures, began to garner media attention, the most high profile of which emanated from the private sector.^78^*The Lancet* was particularly critical and implied that financial motivations for its hasty uptake were in turn connected to the rhetoric of patient prioritisation that surrounded the minimally invasive movement. ‘Laparoscopic surgery has swept the board’ it commented in 1992, ‘launched by Wall Street with barely a glance at clinical trials or orthodox medical conventions, it stands out as the first big patient and technology led revolution in health care’.^79^ Such sentiments positioned minimally invasive procedures as commercially driven and thus, in conflict with the uncompromised scientific objectivity the randomised controlled trial was supposed to represent.^80^

Even more overtly than ESWL, the diffusion of laparoscopic cholecystectomy seemed to suggest that surgical innovations were particularly difficult to assess by the methods that were at the heart of evidence-based medicine, rapidly gaining ground as a movement in the 1990s.^81^ The evidence-based medicine movement had its roots in North America, but was robust in Britain. In 1993 the Cochrane Collaboration had been established, providing a database of randomised controlled trials, enabling meta-analysis of data to take place, while in 1995 the *British Medical Journal* publishing company began issuing the journal *Evidence-Based Medicine*.^82^ If surgeons did not use such methods, they ran the risk of appearing unscientific and out-of-date in their practices. The lack of evidence-based research in surgical innovation was lamented by *Lancet* editor Richard Horton in 1996 who criticised the preference in surgery for the case series rather than the clinical trial.^83^ The haphazard diffusion of minimally invasive or ‘keyhole’ procedures was thus taken to be representative of a wider problem in surgery—which saw innovations like Wickham’s seemingly belie the normal channels of scientific authentication.

## The Society for Minimally Invasive Therapy

In the 1980s a number of different terms were being used to tie together the percutaneous, laparoscopic and radiological procedures that together formed an alternative to open surgery. The result was a fluid and contested nomenclature, which suggested a conceptual instability. Many practitioners used the phrase ‘minimal access surgery’ (MAS), championed by surgeon Alfred Cuschieri, who was pioneering laparoscopic techniques in Dundee.^84^ As we have seen, Sir Lancelot Spratt’s dismissive phrase, ‘keyhole’ surgery, had also made a return at the end of the decade, as it began to be used widely in the media as a description for minimally invasive surgery, although this time usually in a positive rather than derogatory sense.^85^ But ‘minimally invasive’ had long been Wickham’s favoured term, and would be used frequently in the medical sphere to describe procedures deriving from endoscopy, radiology and laparoscopy. Alongside his own innovations, John Wickham was soon leading a wider movement to frame his work as a philosophy of practice rather than an array of techniques specific to one specialty. In 1989 Wickham formed the Society for Minimally Invasive Surgery. The aims of the Society were to ‘develop a closer cooperation between clinicians and manufacturers in research and development; to present papers which offer[ed] practical help to those embarking on minimally invasive surgery; and to introduce courses throughout the world instructing surgeons in the various minimally invasive techniques.’^86^ Surgeons, radiologists and instrument manufacturers from across the globe were invited to form a tripartite organisation. In December of that year, during its inaugural meeting, the Society for Minimally Invasive Surgery became the Society for Minimally Invasive Therapy (SMIT). This change in the name was significant. It was suggested to Wickham at the Society’s inaugural meeting by a participating radiologist who felt it necessary to ensuring that the society was egalitarian rather than one which prioritised its surgical members.^87^ The new name appeared to challenge an established hierarchy of practice which had privileged surgeons as the most authoritative individuals in devising and executing invasive procedures. Underlying the change were also questions of territory which had to be delicately negotiated. Wickham and fellow surgeon and SMIT member John Fitzpatrick expressed anxiety in an article in the *British Journal of Surgery* that surgeons were increasingly losing ground to interventional radiologists by not taking up opportunities to learn minimally invasive techniques to the degree the latter were.^88^ The new name offered an elevated status to radiologists, while supporting the continued claim of surgeons upon the discipline. However, the new term was not without its limitations: Wickham initially worried that it made them sound like ‘a bunch of homoeopathists’.^89^

The Society became instrumental in promoting the idea that minimally invasive therapy offered a radical new vision for the medical profession, a type of practice which blurred the boundaries not just between medical specialties, but between industry and the medical profession as well. In surgery, especially, where instruments, hands, movements and manipulations are inextricably connected, it has long been common for surgeons to collaborate with manufacturers in producing new instruments. Claire L. Jones has examined how nineteenth-century practitioners and product manufacturers frequently interacted through medical trade catalogues, in which surgeons and physicians advertised their own designs and endorsed one another’s products and devices.^90^ In the twentieth-century context this relationship has been well documented by Julie Anderson, Francis Neary and John Pickstone in their study of the history of Total Hip Replacement (THR). As the authors note, John Charnley, the orthopaedic surgeon who initially developed the operation worked in close collaboration with engineers and manufacturers to develop the procedure, which like many of Wickham’s, was heavily dependent on technology.^91^ Similarly, in his study of the introduction and diffusion of the osteosynthesis procedure in the second half of the twentieth century, Thomas Schlich has examined the deeply symbiotic relationship between surgeons, scientists and manufacturers through which the operation was constructed, and the power dynamic inherent to these complex occupational interdependencies.^92^

Where Wickham and the Society for Minimally Invasive Therapy differed was in seeking to openly formalise this non-hierarchical network. In part this was a reflection of Wickham’s own personal and professional experiences; just as he had enjoyed a collaborative working relationship with radiologist Michael Kellett, he had found a similar one with the instrument manufacturer, Stuart Greengrass. Greengrass had joined instrument manufacturers Keymed in 1972 before the company was taken over in 1980 to form Olympus Keymed. Interviewed in 2013, Greengrass recalled the 1980s as a time when medical technology was swiftly developing. He spoke of being ‘immediately engulfed in the tidal wave of endoscopic technology development’, and struck by the ‘beauty and complexity’ of endoscopic instruments.^93^ Like Wickham, Greengrass enthusiastically embraced the possibility of radical change in surgery; ‘that excitement of being able to see inside things without pulling them apart has stayed with me until this day’ he recounted.^94^

There were, however, limitations to Wickham’s vision of a flattened hierarchy. Records of the 84 attendees of the inaugural meeting of SMIT indicate the majority of delegates were surgeons.^95^ It might be assumed that resistance to this tripartite structure would have been greatest from surgeons and radiologists, concerned about the implications of commercialism that might come from a formal collaboration between profession and industry. However, it was the manufacturers who were initially most cautious about becoming involved with the Society. This Wickham attributed to concerns on the part of companies that presenting to the society would result in industrial secrets being divulged to competitors.^96^

Through the Society Wickham was able to stress the impact minimally invasive therapy could have on the organisation of the profession as a whole. Procedures like extracorporeal shock wave lithotripsy (centred upon radiological imaging) or laparoscopic operations (where cameras and complex new instruments were pivotal) seemed to suggest that technological prowess rather than dexterity would be of ever more importance to the practitioners’ skill-set and experiences. As Schlich has highlighted, definitions of surgical skill are dependent on social context.^97^ The move towards more technologically oriented surgery suggested a transition in understandings of skill which reflected and reinforced the value ascribed to sophisticated technological solutions to illness in the late twentieth century.

**Figure 1:**
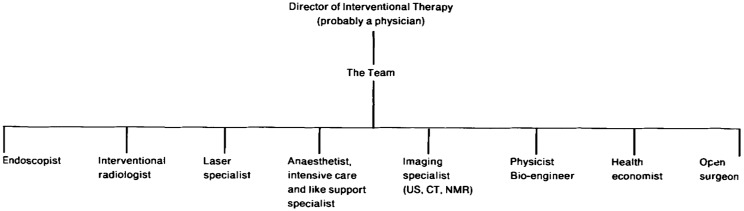
A table from John Wickham’s introductory editorial to the first issue of *Minimally Invasive Therapy* in 1991. In it Wickham envisioned the future organisational structure of ‘interventional therapy’, with the role of open surgeon reduced to a comparatively minor position of equal status to endoscopists, radiologists and others (reproduced with the permission of the author)

As Wickham saw it, rather than increase the authority of surgeons this shift might erode it, dissolving traditional divisions between the surgical and the non-surgical and transforming medicine into an array of distinct specialists, accorded equal status, although interestingly Wickham at the time predicted the overall director would likely be a physician (that is a doctor who practised medicine rather than surgery), maintaining the traditional notion in medicine that the physician heads the medical hierarchy (Figure 1).The implications for surgeons, Wickham predicted, would be profound. As he put it in the first edition of the Society’s journal in 1991, ‘the surgeon is almost destined to become a technologist despite the hope that he might remain the all-encompassing “physician and philosopher” who operates’. The effect of this upon the structure of medical services would also be far-reaching, with Wickham foreseeing an increase in day surgery meaning that surgical patients’ requirements would change. Large hotel-style hospitals, designed for long-term stay would no longer be so frequently required, while areas with good car parking and rapid transport facilities would become essential.^98^

This transformation of minimally invasive techniques into a framework for medicine as a whole would be mined for its political potency. Wickham’s vision spoke to long-held aspirations within medicine for organisational efficiency.^99^ It also appealed to the increasing push for patient empowerment. But the prospect of a drastically reduced convalescence period in hospital fitted especially closely with the ambitions of the prevailing Conservative government, under which patient care was being shifted ever further away from the hospital setting and towards primary and community care.^100^ In 1994 Health Secretary Virginia Bottomley confidently proclaimed that hospital beds would be cut by 40 per cent by 2002, the politician citing laparoscopic surgery as a major contributing factor to this.^101^

In laying out his vision Wickham was controversial—predicting the demise of surgery as a distinct specialty and the transformation of surgeons into technicians. This shift, particularly the joining of radiologists and surgeons, has often been characterised as something akin to a hostile takeover of surgery by radiologists. Examining the North American experience, Lawrence Rosenberg and Thomas Schlich have argued that surgery today may be considered ‘a victim of technological progress’ and have framed the minimally invasive movement as resting upon the decline of surgery in the face of competition—in terms of both status and profit—from radiologists and physicians.^102^ James R. Zetka has similarly shown this was the case in his sociological study of the introduction of endoscopic and radiological techniques into North American gastroenterology and general surgery. Zetka cites in particular the ‘market threat’ that radiologists were seen to pose.^103^ Certainly there were elements of this at work in the British (and indeed European) context too; as Wickham and Fitzpatrick’s article described above suggests, there were fears that general surgeons in Britain would lose operations if they did not adapt. Furthermore, practitioners were not averse to using technologically deterministic rhetoric to buttress their case for minimally invasive practices, as revealed in John Wickham’s appeals to the DHSS about the lithotripter, where he had tried to emphasise that ‘these machines are coming!’. But the Society for Minimally Invasive Therapy—initiated by a surgeon and with a popular membership among surgeons—shows that one must be wary of framing the transition to minimally invasive practice as something that was simply ‘done’ to surgery by exterior medical and non-medical forces. In Britain at least it was surgeons who initiated much of the work in the field, as a means of both maintaining a share in medical practice, but also as a way of facilitating collaboration.

## Conclusion

John Wickham’s minimally invasive therapy project provides an important window on to surgical innovation in twentieth-century Britain. In particular, it points to three aspects that were central to the innovation and, more broadly, to the practice of surgery at this time: surgical invasiveness, the construction of evidence and the professional status of the surgeon.

Minimally invasive therapy was driven by an impetus in British surgery during the second half of the twentieth century to minimise the surgical incision and attendant iatrogenic damage. But it also reflected long-held concerns within the profession about the extent to which surgical invasiveness was necessary or acceptable. These concerns formed the foundation for Wickham’s claim that patients inherently preferred the least invasive procedure that it was possible for them to have, and that, ‘the patient was always right’. The minimally invasive movement also brought into focus long-standing issues regarding the construction of evidence in surgery. As David Jones has already elucidated, surgeons were often resistant to the use of randomised controlled trials for innovations they felt were already self-evidently successful. The controversies over Wickham’s lithotripsy practice and the subsequent rapid diffusion of laparoscopic cholecystectomy, demonstrate the extent to which this was also the case in the British context. It is a debate which continues today. While the majority of new operations continue to be innovated outside of clinical trials and the regulatory framework they give rise to, trials are becoming an ever more common part of surgical research. Initiatives such as IDEAL for example, an international collaboration between surgeons, statisticians and others, seeks to address the challenges of undertaking randomised controlled trials.^104^

While such initiatives premise regulation as key to improving the quality of surgical innovation, historicising the work of Wickham and his colleagues shows the diverse ways in which the framework for innovation and surgical evidence has been imagined. One must, of course, be wary of making retroactive judgements pertaining to a remembered ‘golden age’ of surgical innovation. Innovation is historically constituted and is defined and read differently depending on the context of time and place. But it is striking that our interviewees almost universally cited a sense of professional freedom during the time most of these innovations occurred—between the late 1970s and early 1990s—in which they felt able to try out new procedures. In comparing this era to the present day, most interviewees felt strongly that Wickham and Kellett’s independent introduction of percutaneous nephrolithotomy would have been considered unacceptable in today’s medico-ethical culture. There was an acknowledgement, however, that a reduction in innovation was ultimately a trade-off in exchange for improved and more informed patient care. As Michael Kellett succinctly put it, the benefits of the ‘old’ way were that:

You [got] to develop something very quickly and be a fantastic help. The losses are you may traumatise the patient and I’m sure some people have been … we know from the literature … but luckily we’ve not had any serious problems. Protecting the patient at the same time delays progression … but you can’t go back now … you can’t change.^105^

Finally, the case of minimally invasive therapy points to the complex and shifting professional dynamics of twentieth-century surgery, much of which directly related to the innovations occurring around the minimally invasive movement. The apparent triumph of ‘keyhole’ surgery seemed to herald the continued—perhaps even increased—dominance of surgeons in the management of the body, which had been established in the late nineteenth century. The success of the minimally invasive approach promised a less fearsome, more patient-friendly form of surgery. However, the movement also pointed to the increasingly blurred boundaries between surgery and other disciplines. While this caused concern about whether surgeons would be able to maintain professional dominance, organisations like the Society for Minimally Invasive Therapy also encouraged this fluidity and interdisciplinarity.

We have argued in this paper that a detailed study of a group of professionals who made a significant contribution to a radical shift in surgery can address historical developments from multiple perspectives. We have shown that minimally invasive therapy was complex, contested and politically charged. By embedding the experiences of Wickham and his contemporaries within a longer historical view of surgical practice, invasiveness and innovation, the conditions for the emergence of the minimally invasive movement can be seen more readily and more clearly, as can the technical, ethical and professional questions it raised, which are only beginning to receive historical attention.

## Footnotes

1 In particular, the term has increasingly become a by-word for economic growth. Since the 1960s use of the term ‘innovation’ in UK parliamentary debate has also greatly increased, the term spreading beyond science and technology to be used in a range of different political arenas. Lew Perren and Jonathan Sapsed, ‘Innovation as Politics: The Rise and Reshaping of Innovation in UK Parliamentary Discourse 1960–2005’, *Research Policy*, 2013, 42, 1815–28.

2 John V. Pickstone, ed., *Medical Innovations in Historical Perspective* (Basingstoke: Macmillan, 1992); Ilana Löwy, ed., *Medicine and Change: Historical and Sociological Studies of Medical Innovation* (Montrouge: John Libbey Eurotext, 1993); Thomas Schlich and Ulrich Tröhler, eds, *The Risks of Medical Innovation: Risk Perception and Assessment in Historical Context* (Abingdon & New York: Routledge, 2006).

3 While practitioners had differing opinions of the relative usefulness of each of these terms, for the sake of consistency we use the terms ‘minimally invasive’ and ‘minimally invasive therapy’ throughout. J. E. A. Wickham, ‘Introduction’, *British Medical Bulletin*, 1986, 42, 221–2, 222.

4 One example, which will be discussed later is cholecystectomy for gallstones. See Michael J. Mack, ‘Minimally Invasive and Robotic Surgery’, *Journal of the American Medical Association*, 2001, 285, 568–72, 568.

5 Roger Kneebone and Abigail Woods, ‘Bringing Surgical History to Life’, *British Medical Journal*, 2012, 345, e8135.

6 A set of initial individual interviews with eighteen surgeons, anaesthetists, nurses and others were conducted from 2011 onward. This amounted to 22.8 hours of interview time. Each interview was replayed in detail and summarised following British Library Oral History guidelines. A further set of individual interviews were carried out with John Wickham between 2013 and 2015 (recorded on audio and video during a total of 4.5 days of interviewing). Six full scale simulation-based re-enactments took place between 2011 and 2016 with three different surgical teams. They were each followed by group interviews (around 2 hours per session), also recorded using audio and video. Three further group discussions, all including John Wickham and Michael Kellett, as well as numerous individual interviews were then undertaken to develop a broader historical perspective on minimally invasive therapy.

7 Grzegorz S. Lityński, *Highlights in the History of Laparoscopy: The Development of Laparoscopic Techniques—a Cumulative Effort of Internists, Gynecologists, and Surgeons* (Frankfurt, Barbara Bernert Verlag, 1996); Lawrence Rosenberg and Thomas Schlich, ‘Twenty-First Century Surgery: Have we Entered Uncharted Waters?’ *Bulletin of the American College of Surgeons*, 2012, 97, 6–11.

8 Rosenberg and Schlich focus on North America, while Lityński takes a broad, international perspective.

9 Peter Stanley, *For Fear of Pain: British Surgery 1790–1850* (Rodopi: Amsterdam, 2003), 74. Stanley cautions against the popular viewpoint that only a small range of operations were performed before the introduction of anaesthesia, *ibid*., 73–4. See also Sally Frampton on the use of abdominal surgery in the early nineteenth century: Sally Frampton, ‘Patents, Priority Disputes and the Value of Credit’, *Medical History*, 2011, 55, 319–24, esp. f.11

10 In 1886 John Erichsen, surgeon at University College Hospital, proclaimed that ‘that the final limits of surgery have been reached in the direction of all that is manipulative and mechanical there can be little doubt’. John Eric Erichsen, ‘An Address Delivered at the Opening of the Section of Surgery’, *British Medical Journal*, 1886, 2 (1337), 314–16, 314.

11 P. P. Staple, ‘John Hunter: A Copy of Notes Taken at his Lectures on Surgery’, 2, 1787, Western Manuscripts MS5598, Wellcome Library, London.

12 Owsei Temkin, *The Double Face of Janus and Other Essays in the History of Medicine* (Baltimore: Johns Hopkins University Press, 1977), 491.

13 The question of whether gynaecological surgery had descended into ‘operative mania’ was hotly debated in the late nineteenth-century medical press. Ornella Moscucci, *The Science of Woman: Gynaecology and Gender in England 1800–1929* (Cambridge: Cambridge University Press, 1990), 160–4.

14 William P. Swain, ‘Transactions of Branches: On Recent Improvements in Surgery’, *British Medical Journal*, 1866, 2 (298), 303–5, 304.

15 Charles Clay, ‘On Ovariotomy and Ovariotomists’, *The Lancet*, 1865, 85, 226–8; 227.

16 The phrase was attributed to the surgeon Robert Liston by the obstetrician Robert Lee in his 1853 publication, *Clinical Reports of Ovarian and Uterine Diseases* (London: John Churchill, 1853), 83.

17 Thomas Schlich, ‘“The Days of Brilliancy are Past”: Skill, Styles and the Changing Rules of Surgical Performance, ca. 1820–1920’, *Medical History*, 2015, 59, 379–403, 393; David Barrow, ‘How the Refinements of Abdominal Surgery Have Influenced General Surgery’, *Transactions of the American Association of Obstetricians and Gynecologists*, 1889, 2, 92–9; 92. As quoted in Schlich.

18
*Doctor in the House*, directed by Ralph Thomas (1954: London, ITV Studios Home Entertainment, 2002), DVD.

19 In cholecystectomy, for example, apart from the introduction of operative cholangiography in the late 1930s (where an x-ray is taken of the bile duct during the operation), the method of performing the operation remained virtually unchanged during this period. Roger Kneebone and Abigail Woods, ‘Bringing Surgical History to Life’, e8135.

20 J. W. Kennedy, ‘Tragedies of the Abdominal Incision’, *American Journal of Surgery*, 1934, 25, 512–20, 513.

21 A. H. Southam, ‘A Comparative Study of Abdominal Incisions’, *British Medical Journal*, 1924, 1 (3299), 513–15.

22 Alfred Pearce Gould, ‘A Clinical Lecture on the Surgery of the Abdominal Wall’, *British Medical Journal*, 1911, 2 (2658), 1517–20, 1517.

23 Interview with Chris Russell, 22 January 2014; ‘WS’, ‘Obituary: CJB Murray’, *British Medical Journal*, 1991, 302 (6792), 1598. The obituary noted that Murray ‘expected his junior staff to carry out his instructions to the last detail’.

24
*Ibid*. American researchers N. Henry Moss and F. Curtis Dohan found that the Philadelphia-based practitioners in their study recommended a convalescence period (i.e. time between the operation and returning to work) of anything between 23 and 102 days for cholecystectomy. N. Henry Moss and F. Curtis Dohan, ‘Surgical Convalescence: When Does it End?’, *Annals of the New York Academy of Sciences*, 1958, 73, 455–64, 462.

25 Moss and Dohan, ‘Surgical Convalescence’, 455.

26 M. A. Heasman, ‘How Long in Hospital? A Study in Variation in Duration of Stay for Two Common Surgical Conditions’, *The Lancet*, 1964, 284, 539–541; J. E. P. Simpson *et al.*, ‘“Right” Stay in Hospital after Surgery: Randomised Controlled Trial’, *British Medical Journal*, 1977, 1 (6075), 1514–16.

27 J. N. Morris, ‘Uses of Epidemiology’, *British Medical Journal*, 1955, 2, 395–401. See also Shaun Murphy, ‘The Early Days of the MRC Social Medicine Research Unit’, *Social History of Medicine*, 1999, 12 (4936), 389–406.

28 The report was the result of an enquiry into the financial expenditure of the National Health Service in response to concerns from the Treasury about overspending. In fact, the report would assuage these fears, concluding that relative expenditure on the NHS had fallen. Nonetheless it was suggestive of an increasing scrutiny of resource use. Rudolf Klein, *The New Politics of the NHS: From Creation to Reinvention* (Abingdon: Radcliffe Publishing, 2010), 25–6, 26.

29 Klein notes that during a conference in 1968, in honour of the twentieth anniversary of the NHS, Henry Yellowlees, Deputy Chief Medical Officer at the Department of Health, claimed that £2 million a year could be saved if surgeons cut the time of hospital stays for patients with appendicitis. However, Yellowlees emphasised that such changes in practice should not be thrust on surgeons, rather that surgeons should be gently persuaded to keep in mind the costs of long-terms hospital stays. Klein, *The New Politics of the NHS*, 62. Department of Health and Social Security, *National Health Service: Twentieth Anniversary Conference* (London: Her Majesty’s Stationery Office, 1968), 45.

30 Interview with David Rosin, 22 August 2012.

31
*Ibid.*

32
*Ibid.*

33 Barron H. Lerner, *The Breast Cancer Wars: Fear and Hope and the Pursuit of a Cure in Twentieth-Century America* (Oxford: Oxford University Press, 2001), 92; Ilana Löwy, *Preventive Strikes: Women, Precancer, and Prophylactic Surgery* (Baltimore: Johns Hopkins University Press, 2010), 117.

34 Alex Mold, ‘Repositioning the Patient: Patient Organizations, Consumerism, and Autonomy in Britain during the 1960s and 1970s’, *Bulletin of the History of Medicine*, 2013, 87, 225–49.

35 For a biographical account of Wickham see Dominic Hodgson, ‘Standing on the Shoulders of Giants: 3. John Wickham based on an Interview 7/11/2009’, *British Journal of Medical and Surgical Urology*, 2012, 5, 111–14.

36 In the 1950s neurosurgery was still a relatively young specialty. In 1955 the *British Journal of Surgery* lamented the lack of adequate neurosurgical units across the country, suggesting there were limited opportunities for young doctors to gain experience in the area. ‘The Neurosurgical Clinic and the Neurological Laboratories at the Royal Infirmary, Manchester’, *British Journal of Surgery*, 1955, 43, 317–23, 321.

37 Interview with John Wickham, 23 January, 2012.

38
*Ibid.*

39 Shelley Jane Spaner and Garth Loren Warnock, ‘A Brief History of Endoscopy, Laparoscopy, and Laparoscopic Surgery’, *Journal of Laparoendoscopic and Advanced Surgical Techniques*, 1997, 7, 369–73, 371.

40 Alexandros Polychronidis *et al.*, ‘Twenty Years of Laparoscopic Cholecystectomy: Philippe Mouret—March 17, 1987’, *Journal of the Society of Laparoendoscopic Surgeons*, 2008, 12, 109–11, 109.

41 For more on the impact of video laparoscopy see James R. Zetka, *Surgeons and the Scope* (Ithaca, NY: ILR Press, 2003), esp.3.

42 It was usually limited to angioplasty, a procedure where stents are inserted to dilate blocked arteries.

43 In his study of hospitals in early twentieth-century America, Joel Howell emphasises that the organisation of the physical space of hospitals is pivotal in the formation of new working practices. Joel D. Howell, *Technology in the Hospital: Transforming Patient Care in the Early Twentieth Century* (Baltimore and London: Johns Hopkins University, 1995), 234–6.

44 Interview with Michael Kellett, 5 December, 2013.

45 P. Alken *et al.*, ‘Percutaneous Stone Manipulation’, *Journal of Urology*, 1981, 125, 463–6.

46 J. E. A. Wickham and M. J. Kellett ‘Percutaneous Nephrolithotomy’, *British Medical Journal*, 1981, 283 (6306), 1571–2.

47 Asher Shushan *et al.*, ‘How Long Does Laparoscopic Surgery Really Take? Lessons Learned from 1000 Operative Laparoscopies’, *Human Reproduction*, 1999, 14, 39–43, 39.

48 Stanley Joel Reiser, *Technological Medicine: The Changing World of Doctors and Patients* (Cambridge University Press: Cambridge, 2009) 188; Howell, *Technology in the Hospital*, 6.

49 Interview with John Wickham, 23 January 2012.

50 The *Oxford English Dictionary* attributes the first use of this word to an English language translation of Swiss psychiatrist Eugen Bleuler’s *Textbook of Psychiatry* of 1924 in relation to neurotic manifestations in patients that have been caused by doctors. ‘iatrogenic, adj.’, OED Online. June 2015. Oxford University Press <http://ezproxy-prd.bodleian.ox.ac.uk:2355/view/Entry/90705?redirectedFrom=iatrogenic&>, last accessed 15 July 2015; Eugen Bleuler, *Textbook of Psychiatry*, A. A. Brill (trans), (London: G. Allen & Unwin, 1924), 502.

51 Ivan Illich, *Limits to Medicine: Medical Nemesis: The Expropriation of Health* (New York: Marion Boyars, 1976), 21.

52 J. E. A. Wickham, ‘The New Surgery’, *British Medical Journal*, 1987, 295 (6613), 1581–2, 1581.

53 Mold, ‘Repositioning the Patient’, 225–49.

54 Wickham was unusual in that he preferred to look directly down an endoscope. The majority of surgeons used cameras.

55 Interview with John Wickham, 31 May 2013. Available to watch at: <https://vimeo.com/122100827>, last accessed 8 July 2015. See also J. E. A. Wickham, ‘Editorial’, *Minimally Invasive Therapy*, 1991, 1, 1–5, 5.

56 James Greig Smith, *Abdominal Surgery* (London: Churchill, 1891), 53.

57 Christopher Lawrence, ‘Medical Minds, Surgical Bodies’, in Christopher Lawrence and Steven Shapin, eds, *Science Incarnate: Historical Embodiments of Natural Knowledge* (Chicago & London: University of Chicago Press, 1998), 156–201, 183.

58 Thomas Schlich, ‘Negotiating Technologies in Surgery: The Controversy about Surgical Gloves in the 1890s’, *Bulletin of the History of Medicine*, 2013, 87, 170–97, 182.

59 C. H. Chaussy, Walter Brendel and E. Schmiedt, ‘Extracorporeally Induced Destruction of Kidney Stones by Shock Waves’, *The Lancet*, 1980, 316, 1265–8, 1267.

60 Charles Webster, *The National Health Service: A Political History* (Oxford: Oxford University Press, 2002), 66; Clifford Williamson, ‘The Quiet Time? Pay-beds and Private Practice in the National Health Service: 1948–1970’, *Social History of Medicine*, 2015, 28, 576–95, 588.

61 Webster, *The National Health Service*, 66.

62 Martin Gorsky, ‘“Searching for the People in Charge”: Appraising the 1983 Griffiths NHS Management Inquiry’, *Medical History*, 2013, 57, 87–107, 90.

63 Mike Boyd, ‘PPP Schemes Can Be Dated Back to the Mid-80s’, *Health Service Journal*, 2001, 111, 18. Parliamentary records show that the lithotripter installation at St Thomas’ engendered debate in the House of Commons on numerous occasions regarding the nature of the deal between the NHS and BUPA and what Labour MPs saw as the creeping privatisation of health services. See for example: ‘St. Thomas’s Hospital (Lithotripter)’, HC Deb 13 April 1984 vol. 58, cc411–2W.

64 Interview with John Wickham, 23 January, 2012.

65 J. E. A. Wickham *et al.*, ‘Extracorporeal Shock Wave Lithotripsy: The First 50 Patients Treated in Britain’, *British Medical Journal,* 1985, 290 (6476), 1188–9.

66 E. L. H. Palfrey *et al*., ‘Report on the First 1000 Patients Treated at St.Thomas’ Hospital by Extracorporeal Shockwave Lithotripsy’, *British Journal of Urology*, 1986, 58 (6524), 573–7.

67 C. R. Charig *et al.*, ‘Comparison of Treatment of Renal Calculi by Open Surgery, Percutaneous Nephrolithotomy, and Extracorporeal Shockwave Lithotripsy’, *British Medical Journal*, 1986, 292, 879–82.

68 S. Challah and N. B. Mays, ‘The Randomised Controlled Trial in the Evaluation of New Technology: A Case Study’, *British Medical Journal*, 1986, 292 (6524), 877–9, 877.

69 Harry Marks, *The Progress of Experiment: Science and Therapeutic Reform in the United States, 1900–1990* (Cambridge: Cambridge University Press).

70 Gerald Kutcher, *Contested Medicine: Cancer Research and the Military* (Chicago and London: University of Chicago Press, 2009), 23.

71 David S. Jones, ‘Visions of a Cure: Visualization, Clinical Trials, and Controversies in Cardiac Therapeutics, 1968–1998’, *Isis*, 2000, 91, 504–41, 523.

72 Jones, ‘Visions of a Cure’, 530.

73 Benjamin Freedman, ‘Equipoise and the Ethics of Clinical Research’, *New England Journal of Medicine*, 1987, 317, 141–5.

74 J. E. A. Wickham, ‘Stones, Lithotripters, Trials and Arguments’, *British Medical Journal*, 1986, 292 (6528), 1134–5, 1134.

75 Interview with John Wickham, 23 January 2012.

76 Interview with Michael Kellett, 5 December 2013.

77 Interview with Chris Russell, 22 January 2014; G. S. Litynski, ‘Erich Mühe and the Rejection of Laparoscopic Cholecystectomy (1985): A Surgeon Ahead of His Time’, *Journal of the Society of Laparoendoscopic Surgeons*, 1998, 2, 341–6, 342.

78 This was the case of Ruth Silverman, who died in June 1992 following an operation for bowel obstruction at a private hospital. The operation had been unexpectedly switched from an open procedure to a laparoscopic one. The patient died nine days later after developing peritonitis, septicaemia and a brain haemorrhage, leading to a temporary moratorium on laparoscopic procedures at the hospital. Sharon Kingman, ‘Private Hospitals Ban Laparoscopic Surgery’, *British Medical Journal*, 1993, 306 (6887), 1227; Clare Dyer, ‘Husband Wins Compensation after Laparoscopy Death’, *British Medical Journal*, 1995, 310 (6979), 551.

79 ‘Colons and Keyholes’ *The Lancet*, 1992, 340, 824–5, 824.

80 Jones, ‘Visions of a Cure’, 521. See also Marks, *The Progress of Experiment*, 25.

81 The term ‘evidence-based’ medicine was coined in 1990 by Canadian physician Gordon Guyatt. As Guyatt defined it in 1992, ‘evidence-based medicine de-emphasizes intuition, unsystematic clinical experience, and pathophysiologic rationale as sufficient grounds for clinical decision making and stresses the examination of evidence from clinical research’. Gordon Guyatt *et al.*, Evidence-Based Medicine: A New Approach to Teaching the Practice of Medicine’, *Journal of the American Medical Association*, 1992, 268, 2420–5, 2420.

82 Jeanne Daly, *Evidence-Based Medicine and the Search for a Science of Clinical Care* (Berkeley and London: University of California Press, 2005), 3. The collaboration was headed up by Oxford based researcher Iain Chalmers.

83 Richard Horton, ‘Editorial: Surgical Research or Comic Opera: Question but few Answers’, *The Lancet*, 347, 984–5.

84 Alfred Cuschieri, ‘“A Rose by any other Name …” Minimal Access or Minimally Invasive Surgery?’ *Surgical Endoscopy*, 1992, 6, 214.

85 The revival and popularisation of the phrase appears to have taken place when it was used during an episode of the BBC1 popular science program *Q.E.D.* screened in 1989 which focused on John Wickham. *Q.E.D.* producer Fiona Holmes decided to use the Lancelot Spratt ‘keyhole’ scene from *Doctor in the House* as a pre-title sequence and subsequently named the program ‘Keyhole Surgery’. With thanks to Fiona Holmes for this additional information.

86 J. E. A. Wickham and J. M. Fitzpatrick, ‘Minimally Invasive Surgery’, *British Journal of Surgery*, 1990, 77, 721–2, 722.

87 Interview with John Wickham, 23 January 2012.

88 Wickham and Fitzpatrick, ‘Minimally Invasive Surgery’, 722.

89 Interview with John Wickham, 23 January 2012.

90 Claire L. Jones, ‘(Re-)reading Medical Trade Catalogs: The Uses of Professional Advertising in British Medical Practice, 1870–1914’, *Bulletin of the History of Medicine*, 2012, 86, 361.

91 Julie Anderson, Francis Neary and John V. Pickstone, *Surgeons, Manufacturers and Patients: A Transatlantic History of Total Hip Replacement* (Basingstoke: Palgrave Macmillan, 2007), 50; as quoted from John Charnley, ‘Surgeon and Engineer’, *The Lancet*, 1961, 277, 325–6, 326.

92 Thomas Schlich, *Surgery, Science and Industry: A Revolution in Fracture Care, 1950s–1990s* (Basingstoke: Palgrave Macmillan, 2002), 46–64.

93 Interview with Stuart Greengrass, 6 December 2013.

94
*Ibid.*

95 Delegate list, inaugural meeting of the Society for Minimally Invasive Surgery, 11–12 December 1989. In the authors’ possession. With thanks to John Wickham for this additional information.

96 Interview with Stuart Greengrass and John Wickham, 6 December 2013.

97 Schlich, ‘“The Days of Brilliancy Are Past”’, 380.

98 Wickham, ‘Editorial’, 4.

99 Steve Sturdy and Roger Cooter, ‘Science, Scientific Management, and the Transformation of Medicine in Britain c.1870–1950’, *History of Science*, 1988, 36, 421–66.

100 The 1990 National Health Service and Community Care Act dramatically shifted power to primary care. It allowed GPs to manage their own budgets and pick and choose competing hospital and outpatient services for their patients. Angela Coulter, ‘Shifting the Balance from Secondary to Primary Care’, *British Medical Journal*, 1995, 311 (7018), 1447–8; Klein, *The New Politics of the NHS*, 174.

101 Celia Hall, ‘A Revolution in Health Care’ *The* Independent, 23 June 1994, <http://www.independent.co.uk/news/a-revolution-in-health-care-1424427.html>, last accessed 15 July 2015.

102 Rosenberg and Schlich, ‘Twenty-First Century Surgery’, 7.

103 Zetka, *Surgeons and the Scope*, 123.

104 <http://www.ideal-collaboration.net/about-ideal/>, last accessed 11 May 2016.

105 Interview with Michael Kellett, 5 December, 2013. In 2012 the conservative peer Maurice Saatchi introduced the Medical Innovation Bill into Parliament. It proposed greater protection for doctors using new treatments which had yet to be verified or validated on an evidence-based level. While the Bill was vetoed in the House of Commons, it raised afresh debates about the extent to which innovation could be undertaken in medical practice. Rob Home *et al.*, ‘A New Social Contract for Medical Innovation’, *The Lancet*, 2015, 385, 1153–4.

